# Antimicrobial Resistance and Increased Virulence of *Salmonella*

**DOI:** 10.3390/microorganisms10091829

**Published:** 2022-09-14

**Authors:** Bijay K. Khajanchi, Steven L. Foley

**Affiliations:** Division of Microbiology, National Center for Toxicological Research, U. S. Food and Drug Administration, Jefferson, AR 72079, USA

This special issue of Microorganisms highlights the importance of antimicrobial resistance (AMR) and increased virulence of *Salmonella* with multiple research papers. Key to the increase in AMR and virulence are plasmids, whose importance is briefly discussed in this editorial. The acquisition of various large plasmids has impacted the global epidemiology and cross-country dissemination of *Salmonella enterica* strains. Plasmids are extrachromosomal, generally circular DNA molecules that often contain genes that provide bacteria several biological functions including virulence and AMR [1]. Some of the *Salmonella* large plasmids possess multiple AMR genes and virulence factors; hence, co-selection of AMR and virulence properties yield potentially more dangerous pathogens [2]. Commonly, these plasmids are low copy number and confer minimal fitness costs. The role of some of these virulence-associated plasmids in the dissemination of AMR and increased virulence in food-animal environments and humans are discussed in this special issue.

*Salmonella* virulence plasmids, commonly known as pSV plasmids, are one of the more well-studied plasmids [3,4,5]. pSV plasmids carry the *Salmonella* plasmid virulence (*spv*) operon, a highly conserved 7.8-kb region, harboring several virulence genes promoting intracellular infections in hosts [3]. *spvABCD* genes encode proteins that are translocated into host cells via the type-3 secretion system (T3SS) and modulate host immune responses by several mechanisms, including preventing actin polymerization and down-regulating host immune responses [6,7,8]. The role of *spv* in virulence and pathogenesis during the infection process was delineated using different model infection approaches, such as the subcutaneous mouse model [6,9] and zebrafish model [10].

Another group of plasmids that contribute to virulence are the incompatibility group (Inc) FIB plasmids that are related to the ColV plasmid, and are commonly found in several *Salmonella* serovars including Kentucky, Typhimurium, and Schwarzengrund. In a study by our group, an IncFIB plasmid was transferred to an IncFIB plasmid deficient strain of *S*. *enterica* by conjugation [11]. The transconjugant SE819::IncFIB persisted in human intestinal epithelial (Caco-2) cells at a higher rate than the recipient SE819 [11]. Another study demonstrated that horizontal gene transfer of IncFIB plasmid resulted in the emergence of a dominant avian clonal type of *S*. *enterica* serovar Kentucky [12]. Additionally, their study examined distribution of these plasmids among 902 *Salmonella* isolates from different poultry sources. The IncFIB plasmid was found to occur predominantly in serovar Kentucky (72.9% of isolates tested), followed by Typhimurium (15%) and Heidelberg (1.7%); the latter two serovars are among the most commonly associated with disease in humans [12]. In a recent study, our data showed that IncFIB-containing food and clinical *S*. Schwarzengrund isolates clustered within the same clade, which was separated from the isolates that lacked IncFIB plasmids (unpublished data). These findings suggested that IncFIB containing *S.* Schwarzengrund persist in a food environment and successfully establish infection in human.

Some *S. enterica* strains contain another virulence-associated plasmid, an IncX4-like plasmid that harbors a VirB/D4 type 4 secretion system (T4SS) [13]. The VirB/D4 T4SS helps *Salmonella* survive better inside macrophages and epithelial cells by likely down regulating the host’s innate immune response. In a study, it was shown that multiple *Salmonella* strains contained IncX4-VirB/D4 plasmids isolated from retail meats, food animals, and human patients associated with a disease outbreak [14]. These data indicate that plasmid factors including VirB/D4 T4SS on the IncX4 plasmid likely play a role in the infection process and/or persistence in food-animal-clinical environments leading to pathogen transmission.

The IncI1 plasmids are widely distributed in enteric bacteria, particularly *Salmonella* and *Escherichia coli* from food animal sources, resulting in clinical significance of bacteria carrying this type of plasmid. These plasmids have the potential to carry and horizontally transfer multiple integron-associated AMR genes including *sul1* and *sul2* sulfonamide resistance genes, and the *bla*_CTX_ *bla*_CMY_, *bla*_SHV_, and *bla*_TEM_ genes that encode resistance to multiple cephalosporins among enteric pathogens [15]. In addition to AMR genes, IncI1 plasmids can likely carry virulence-associated genes. We demonstrated that bacteriocins encoded by IncI1 of *Salmonella* inhibit growth of *E. coli* which is likely a beneficial selection advantage in growth competition in certain environment [16].

The recent global emergence of *S*. Infantis has been associated with the acquisition of a unique mega-plasmid known as the plasmid of emerging *S.* Infantis (pESI), that confers multidrug resistance and increased virulence phenotypes [17,18,19]. Despite its large size (280 kb), the pESI plasmid does not appear to show a fitness cost [18]. When birds were infected orally, pESI positive strains showed significantly increased virulence compared to pESI negative strains [20]. Studies demonstrated that pESI also has the potential to transfer resistance and virulence to commensal *E*. *coli* and other pathogenic bacteria in the gut environment [18,21].

As the examples above demonstrate, plasmids are important elements that can impact public health. Many plasmids carry mobile genetic elements (MGEs), such as integrons and insertion sequences (ISs), that facilitate transfer of AMR genes. ISs are the simplest MGEs that generally harbor one or more transposes (*tnp*) genes and are widespread in all domains of life [22]. IS26, an 820 bp DNA segment that encodes a transposase (Tnp26) of 234 amino acids [23], was found to be very critical in the dissemination of multiple antibiotic resistance genes including those found in carbapenems [24]. IS26 is widely spread in many antibiotic-resistant isolates and plays crucial roles in the diversity of the variable regions of different plasmids [25,26]. IS26-mediated gene transfer is usually accomplished by cointegration where transferable IS26 cointegrates with pre-existing IS26 sites [24,27].

It is likely that the widespread use of antibiotics has facilitated the emergence of highly resilient pathogens that pose a threat to public health via co-selection of AMR genes and virulence factors [2]. These plasmids likely contribute increased virulence characteristics to the host bacteria that harbor them. Many of the IncF-type plasmids, including those discussed above, are self-conjugative and play an important role in the dissemination of resistance and virulence through horizontal gene transfer. Some *Salmonella* strains can harbor multiple virulence-associated plasmids and become highly virulent. While some gene transfer mechanisms are known, more research is needed to identify other unknown mechanisms as to how virulence and AMR plasmids disseminate among *Salmonella* and other pathogens. This will likely aid the development of proper intervention strategies to control the spread of these plasmids in pathogens prevailing in the food-animal environment.
